# Tumour cell-derived debris and IgG synergistically promote metastasis of pancreatic cancer by inducing inflammation via tumour-associated macrophages

**DOI:** 10.1038/s41416-019-0595-2

**Published:** 2019-10-07

**Authors:** Qi Chen, Jianxin Wang, Qi Zhang, Jingying Zhang, Yu Lou, Jiaqi Yang, Yiwen Chen, Tao Wei, Jian Zhang, Qihan Fu, Mao Ye, Xiaozhen Zhang, Xiaowei Dang, Tingbo Liang, Xueli Bai

**Affiliations:** 1 Department of Hepatobiliary and Pancreatic Surgery, the First Affiliated Hospital, Zhejiang University School of Medicine, Hangzhou, China; 2 Zhejiang Provincial Key Laboratory of Pancreatic Disease, Hangzhou, China; 3 Zhejiang Provincial Innovation Center for the Study of Pancreatic Disease, Hangzhou, China; 4 Department of General Surgery, the Second Affiliated Hospital, Zhejiang University School of Medicine, Hangzhou, China; 5 Department of Hepatopancreatobiliary Surgery, The First Affiliated Hospital of Zhengzhou University, Zhengzhou, China

**Keywords:** Cancer microenvironment, Cancer microenvironment

## Abstract

**Background:**

The progression and metastasis of pancreatic ductal adenocarcinoma (PDAC) is highly dependent on the tumour microenvironment. Most tumour-associated macrophages (TAMs) are M2 phenotype macrophages, which normally show anti-inflammatory functions in numerous disorders. Previously, we found that alternatively activated macrophages showed pro-inflammatory characteristics upon stimulation with hepatoma cell-derived debris; however, the molecular mechanism was unclear.

**Methods:**

In vitro and in vivo experiments were employed to investigate the molecular mechanism. Using pancreatic cancer cell lines, mouse models and human tissues, we obtained a general picture of tumour cell-derived debris promoting metastasis of pancreatic cancer by inducing inflammation via TAMs.

**Results:**

We showed that M2 macrophage-derived inflammation also exists in PDAC. Debris from PDAC cells induced potent IL-1β release by M2 macrophages via TLR4/TRIF/NF-κB signalling, and this effect was further boosted by IgG that was also derived from PDAC cells. Increased IL-1β promoted epithelial–mesenchymal transition and consequent metastasis of PDAC cells. A selective COX-2 inhibitor, celecoxib, enhanced the anti-tumoural efficacy of gemcitabine.

**Conclusions:**

These data revealed a pro-inflammatory mechanism in PDAC, which indicated that IL-1β and COX-2 could be therapeutic targets of an anti-inflammatory strategy to treat PDAC.

## Background

Pancreatic ductal adenocarcinoma (PDAC) is one of the deadliest cancers because of delayed diagnosis and limited response to current treatment modalities.^1,2^ Despite decades of effort, the 5-year overall survival rate remains <10%, making PDAC one of the leading causes of cancer-related deaths worldwide.^3^ The local inflammatory microenvironment plays key roles in tumour development.^4^ Numerous studies have revealed a variety of mechanisms by which tumours and inflammation interplay with each other.^5^ For PDAC, which is characterised by desmoplasia, the roles of stromal cells in tumour progression are more complicated. Tumour-associated macrophages (TAMs) are essential players in the microenvironment of PDAC and are believed to facilitate tumour progression in a variety of ways.^6^ TAMs frequently show M2 polarisation with anti-inflammatory functions. This immune suppression role of TAMs contributes greatly to their pro-tumoural effects. However, it was revealed recently that alternatively activated macrophages could exert pro-inflammatory functions in certain diseases, such as rheumatoid arthritis. We also revealed that TAMs with typical M2 phenotypic markers and significant interleukin-1β (IL-1β) secretion existed in hepatocellular carcinoma.^7^ These findings suggested that the actual role of TAMs in solid cancers is still perplexing and far from understood.

IL-1β is a classic and potent pro-inflammatory cytokine that is mainly secreted by macrophages. IL-1β induces hypoxia-inducible factor-1α (HIF-1α) overexpression at a very low concentration. Given that overexpression of HIF-1α significantly correlates with poor prognosis in patients with PDAC,^8^ the increased level of IL-1β in PADC is important for tumour progression. However, the origin of IL-1β is confusing because the tumour microenvironment is typically anti-inflammatory and immunosuppressive. We speculated that in PDAC, IL-1β is released by M2 polarised TAMs, in a similar manner to found in liver cancer, and interference with its effects would be beneficial for PDAC control.

Unfortunately, the mechanisms of IL-1β secretion by M2 polarised macrophages are largely unknown, although Fcγ receptor (FcγR) signalling might be critical.^9^ Previously, we demonstrated that certain *O*-glycosylated proteins from the necrotic debris of cancer cells was sufficient to induce pro-inflammatory M2 polarised TAMs;^7^ however, the role of IgG in this process is unclear. This issue is important because various cancers, including PDAC, can produce IgG in a secretory form.^2,10,11^ In the present study, we report that IL-1β signalling, which involves the interaction of tumour cells and M2 polarised TAMs, promotes PDAC progression. Anti-inflammation using a COX-2 inhibitor could counteract the IL-1β-induced pro-tumoural effects and sensitise PDAC to chemotherapy. We also propose a preliminary mechanism by which IgG enhances IL-1β production via M2 polarised TAMs.

## Materials and methods

### Cell culture and reagents

PANC-1, BxPC-3, MIAPACA-2 and ASPC-1 cells were purchased from the American Type Culture Collection (ATCC; Manassas, VA, USA). THP-1 and Raji cells were purchased from the Shanghai Institute for Biological Science (Shanghai, China). All pancreatic cancer cell lines were authenticated by a professional biotechnology company (Genetic Testing Biotechnology, Suzhou, China), and were used within 3 months of resuscitation. The KPC cell line, which was derived from the spontaneous tumour of an LSL-Kras^G12D/+^; LSL-Trp53^R172H/+^; Pdx1-Cre mouse model, was a kind gift from Prof. Raghu Kalluri’s laboratory (Department of Cancer Biology, Division of Science, MD Anderson Cancer Center, Houston, TX, USA). Human peripheral blood mononuclear cells (PBMCs) were obtained from the Zhejiang Provincial Blood Center (Hangzhou, China). THP-1, PANC-1, BxPC-3, ASPC-1, KPC, and Raji cells were cultured in Roswell Park Memorial Institute (RPMI)-1640 medium (Gibco, Thermo Fisher Scientific, Waltham, MA, USA). MIAPACA-2 cells were cultured in Dulbecco’s Modified Eagle’s medium (DMEM) (Gibco). PBMCs were cultured in Iscove’s Modified Dulbecco’s Medium (IMDM) (Gibco). All media were supplemented with 10% fetal bovine serum (FBS; Thermo Fisher Scientific) and 1% penicillin/streptomycin (Genom, Hangzhou, China). For MIAPACA-2 cells, additional horse serum (Gibco) to a final concentration of 2.5% was required. For hypoxia treatment (1% O_2_), the cells were cultured in a sealed hypoxia chamber (Thermal Tech, Orlando, FL, USA). Unless otherwise stated, recombinant human IL-1β (Peprotech, Rocky Hill, NJ, USA) was used at a final concentration of 1 ng/ml. Celecoxib (Pfizer, New York, NY, USA) was used at a dose of 5 µM in PANC-1 and BxPC-3 cells. Gemcitabine (Eli Lilly, Indianapolis, IN, USA) was used for 48 h at doses of 100 nM and 10 nM in PANC-1 and BxPC-3 cells, respectively.

### M2 macrophage differentiation

THP-1 cells and PBMCs were used to generate M2 macrophages. For macrophage differentiation, THP-1 cells were treated overnight with 200 nM phorbol 12-myristate 13-acetate (PMA; Sigma-Aldrich, St. Louis, MO, USA) in RPMI 1640. PBMCs were treated with 30 ng/ml of recombinant human macrophage colony-stimulating factor (M-CSF; Peprotech) for 6 days. Half of the medium was replaced with fresh medium containing M-CSF on day 3. To induce M2 macrophages, THP-1 cells were further treated with 25 ng/ml human interleukin (IL)-4 and 25 ng/ml human IL-13 (both from Peprotech) for 48 h.

### Generation of tumour necrotic debris

To generate pancreatic cancer necrotic debris, BxPC-3 cells were cultured within a 75-cm^2^ flask (Corning, Corning, NY, USA) under hypoxic conditions (1% O_2_) for 48 h after reaching 100% confluence. Necrotic cells and supernatants were then collected and centrifuged at 12,000 rpm for 10 min at 4 °C. The cell debris was washed three times with phosphate-buffered saline (PBS; Genom) to remove soluble components. The debris was resuspended in 1 ml Opti-MEM (Gibco) and stored at −80 °C until use. All samples were tested for the presence of bacterial endotoxin using Limulus Amebocyte Lysate Test (Genscript, Nanjing, China) and only those with bacterial endotoxin <0.02 EU/ml were used for subsequent experiments.

### Debris and IgG stimulation assay

Human IgG (Sigma-Aldrich) was re-suspended in PBS at a final concentration of 100 ng/µl and was stored at −80 °C until use. For the debris and IgG stimulation assay, macrophages were seeded at a density of 20,000 cells per well in a 24-well plate. Debris (15 µl) or 2.5 µl of IgG or both in 500 µl of Opti-MEM was used to stimulate macrophages for 24 h (debris from four BxPC-3 cells per macrophage), followed by treatment with 5 mM adenosine 5′-triphosphate (ATP; Sigma-Aldrich) for another 2 h. The supernatants were collected by centrifugation for further assays.

### Toll like receptor 4 (TLR4) signalling inhibition and FcgR blockade assays

Toll like receptor adaptor molecule 1(TICAM1 or TRIF) and MYD88 innate immune signal transduction adaptor (MYD88) of macrophages were inactivated by incubating with Pepinh-TRIF (5 µM; Invivogen, San Diego, CA, USA) and Pepinh-MyD88 (5 µM; Invivogen), respectively, for 6 h at 37 °C before the cells were subjected to stimuli. FcγRs of macrophages were blocked by incubation with anti-FcγRI (CD64; BD Biosciences, San Diego, CA, USA), anti-FcγRIIa (CD32a; Stem Cell Technologies, Vancouver, Canada), anti-FcγRIIb (CD32b; OmnimAbs, Alhambra, CA, USA), anti-FcγRIII (CD16; BD Biosciences) antibodies, or mouse IgG (Sigma-Aldrich) at 20 mg/ml for 30 min at 37 °C, after which the cells were subjected to stimuli, resulting in a final antibody concentration of 5 mg/ml.

### Transwell and wound healing assays

Both assays were performed as descried previously.^12^ In brief, Transwell chambers with membranes containing 8-µm pores were coated with Matrigel matrix (Corning). Fifty thousand cells were seeded in the upper compartment of the chamber. The cells on the upper side of the membrane were removed after 48 h. Cells invading to the other side of the membrane were fixed and stained using 0.1% crystal violet (Sigma-Aldrich). Stained cells were counted in five random fields at a ×100 magnification. Each assay was performed in triplicate. For the wound healing migration assays, half a million cells were seeded in a six-well plate. A wound of approximately 0.5 mm wide was made using a sterile pipette tip. Floating cells were removed by washing with medium three times, and fresh medium with 1% FBS was then added. Images were acquired at the indicated times. The width of the scratch at time zero and at the final time point were measured and compared. Three independent assays were performed.

### Immunoblotting, immunohistochemistry and ELISA

Immunoblotting and immunohistochemistry were performed as previously described.^12^ In brief, total proteins were extracted using Radioimmunoprecipitation assay (RIPA) lysis buffer containing 1 mM phenylmethylsulfonyl fluoride (PMSF) and separated using sodium dodecylsulfate polyacrylamide gel electrophoresis (SDS-PAGE). After transferring the separated proteins onto polyvinylidene difluoride (PVDF) membranes, the membranes were blocked with 5% non-fat milk and incubated overnight at 4 °C with the indicated primary antibodies, followed by the corresponding secondary antibodies. The primary antibodies used in this study included: anti-glyceraldehyde-3-phosphate dehydrogenase (GAPDH), anti-E-cadherin, anti-vimentin, anti-nuclear factor kappa B (NF-κB), anti-phosphorylated (p)-NF-κB (p65, Ser536), anti-mammalian target of rapamycin (mTOR), anti-p-mTOR (Ser2448), anti-AKT kinase (AKT), anti-p-Akt (Ser473), anti-caspase-3, anti-cleaved caspase-3, anti-HIF-1α, anti-spleen associated tyrosine kinase (SYK), anti-p-SYK (Tyr525/526), anti-cyclooxygenase 2 (COX-2), anti-TLR4, anti-IL-1β and anti-IgG antibodies. The anti-IL-1β and anti-IgG antibodies were purchased from Abcam (Cambridge, MA, USA). The anti-TLR4 antibodies were purchased from Santa Cruz (Santa Cruz, CA, USA). Other antibodies were purchased from Cell Signaling Technology (Danvers, MA, USA). Secondary antibodies were purchased from Hua’an Biotech (Hangzhou, China). The immunoreactive proteins on the membranes were then visualised using a ChemiDoc XRS System (Bio-Rad Laboratories, Hercules, CA, USA).

For immunochemistry, formalin-fixed paraffin-embedded PDAC tissue samples were cut into 5-μm sections and subjected to heat-induced antigen retrieval. The following antibodies (all from Cell Signaling Technology) were used: anti-IgG (1:100), anti-E-cadherin (1:100), anti-vimentin (1:100), anti-CD68 (1:200), anti-CD206 (mannose receptor C-type 1) (1:100), anti-marker of proliferation Ki-67 (Ki67) (1:200), and anti-caspase-3 (1:200) antibodies. The slides were then incubated with horseradish peroxidase (HRP)-conjugated antibodies against rabbit or mouse IgG using a Histostain-Plus Kit (ZSGB-BIO, Beijing, China). The slides were counterstained with haematoxylin and checked under a microscope (Leica, Wetzlar, Germany). Negative controls were performed without the application of primary antibodies.

For enzyme-linked immunosorbent assay (ELISA), supernatants were collected by centrifugation and stored at −80 °C. Concentrations of IL-1β were detected using specific ELISA kits (Dakewei, Shenzheng, China or Biolegend, San Diego, CA, USA) according to the manufacturer’s instructions.

### Reverse transcription polymerase chain reaction (RT-PCR) and qRT-PCR

Total RNA was extracted using the Trizol LS Reagent (Invitrogen, Carlsbad, CA, USA), and was applied for cDNA generation using SuperScript III Reverse Transcriptase (Invitrogen). Premix Taq Polymerase (Takara Bio, Dalian, China) was used for amplification of Igκ, Igλ and Ig gene variable regions. Nested PCR and touchdown PCR reactions were performed to detect target genes, as previously described.^2^ The primers used are listed in Supplementary Table 1. The final products were analysed using agarose gel electrophoresis. Raji cells were used as a positive control.

For quantitative real time reverse transcription PCR (qRT-PCR), cDNA was acquired using PrimeScript RT reagent Kit (Takara), and cDNA was applied for the qPCR reaction using SYBR Green Real-Time PCR Master Mix (Takara) and Applied Biosystems 7500 Fast Real-Time PCR System (Applied Biosystems, Foster City, CA, USA) according to the manufacturer’s instructions, and the comparative CT method was used for analysis. All primers were synthesised by Sangon Biotech Inc. (Shanghai, China) and Xiangyin Inc. (Shanghai, China) (Supplementary Table 2).

### Flow cytometry

Tumour tissues were washed with PBS three times, cut into small pieces with scissors, and then minced completely using sterile scalpel blades. The minced tumour pieces were incubated with ultrapure collagenase IV (Worthington Biochemicals, Freehold, NJ, USA) at 37 °C for 1 h, with manual dissociation every 15 to 20 min by pipetting. Single cells were filtered through a 70-μm nylon mesh (Corning) and washed with PBS supplemented with 2% FBS. Cells were stained with specific antibodies according to the manufacturer’s instructions. The antibodies including anti-mouse CD45 (HI30), CD4 (RM4-5), CD11b (ICRF44), GR-1 (RB6-8C5), FOXP3 (236 A/E7), anti-human CD68 (Y1/82 A), CD163 (RM3/1), CD206 (15-2), mouse Fc block (2.4G2), and human Fc block (TruStain FcX) were purchased from BD Biosciences or Biolegend. Isotype controls were used for all staining. Flow cytometry was performed on a BD FACS Canto-II Flow Cytometer (BD Biosciences), and data were analysed using FlowJo software (version 10.6, Ashland, OR).

### Mouse models and experiments

Male C57BL/6 mice (6-weeks-old) were purchased from the Shanghai Experimental Animal Center (Shanghai, China), and were feeding in SPF facility. Experiments were performed according to the ethical guidelines of the Ethics Committee of our hospital. KPC tumour cell inoculation was performed on day 0. Briefly, mice were anaesthetised with pentobarbital sodium (35 mg/kg, i.p.) and the middle abdominal incision is made. The pancreas is eviscerated from the mouse and half a million KPC cells were re-suspended in 25 μl of PBS and implanted orthotopically into the capsule of the pancreas. The peritoneum and the skin were then sutured. For the IL-1β treatment assay, mice were randomly allocated to two groups at two weeks after inoculation. In the experiment group, murine IL-1β (Peprotech) were administered intraperitoneally at 500 ng (in 100 μl PBS) per dose on days 14, 21, 28, 35 and 42. The control group received an equal volume of vehicle. Mice were monitored daily and euthanised humanely by CO2 inhalation on day 49, and tumours were harvested for further evaluation. For the celecoxib or gemcitabine treatment assay, mice were randomly divided to four groups at 3 weeks after inoculation. In the celecoxib group, the celecoxib compound was dissolved into PBS and given to the tumour-bearing mice by oral gavage at 50 mg/kg starting on day 21 and continuing every other day for a total of seven doses. In the gemcitabine group, gemcitabine (14.25 mg/kg) was given intraperitoneally every other day on Day 21 following surgery for two weeks. The combination group was treated with both gemcitabine and celecoxib every other day using the same doses and approaches as the celecoxib and gemcitabine groups. The control group was treated with an equal amount of vehicle. Mice were euthanised humanely by CO2 inhalation after two weeks of treatment and tumour samples were harvested for further study.

### Human tissue acquisition

Human tumour samples were obtained from patients with PDAC who underwent curative resection between 2013 and 2016 in our department. Written informed consent was obtained from all patients. The protocol of this study was approved and supervised by the Ethics Committee of our hospital.

## Statistical analysis

The data were analysed using SPSS (version 24, IBM Corp., Armonk, NY, USA) and GraphPad Prism 6 (GraphPad Software, San Diego, CA, USA). Data are presented as mean ± standard deviation (SD). Continuous variables were analysed using an unpaired Student’s *t*-test to compare between two groups, or one-way analysis of variance (ANOVA) for multiple groups. Categorical variables were compared using a *chi*-squared test or Fisher exact test, as appropriate. A *p* value < 0.05 was considered statistically significant.

## Results

### M2 polarised macrophages and their pro-inflammatory function in PDAC

**Fig. 1:**
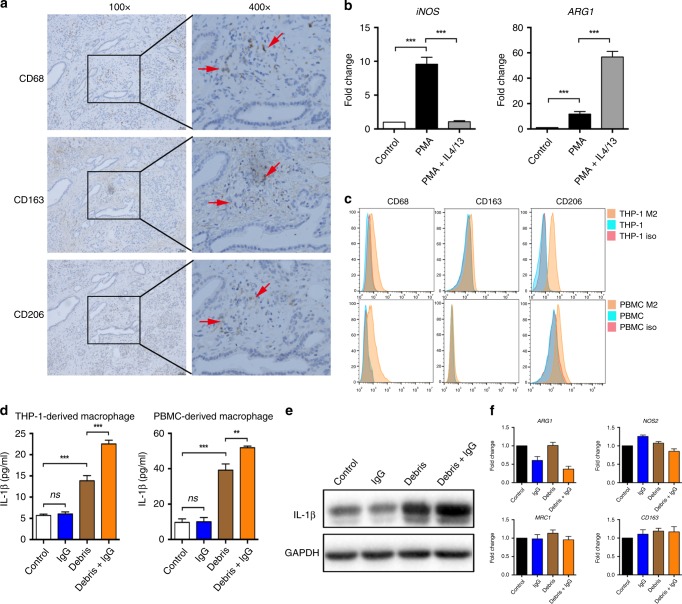
IL-1β released from alternatively activated macrophages stimulated by necrotic cancer cell debris. **a** CD68^+^, CD163^+^ and CD206^+^ tumour-associated macrophages in the human PDAC samples. Scale bar represents 50 µm. **b** Changes in the mRNA levels of *NOS2* and *ARG1* indicated M2 polarisation of THP-1-derived macrophages in the presence of IL-4/IL-13. **c** Changes in CD68, CD163 and CD206 expression levels indicated M2 polarisation of THP-1-derived macrophages in the presence of PMA and IL-4/IL-13 (superior panel), or M2 polarisation of PBMC-derived macrophages in the presence of M-CSF (inferior panel). **d** The secreted IL-1β level in M2 polarised macrophages derived from THP-1 (left panel) and human PBMCs (right panel) in the presence of IgG and/or BxPC-3 debris. **e** Immunoblotting showing enhanced IL-1β levels in the presence of debris stimulation and IgG. **f** Levels of macrophage phenotypic markers including ARG1, NOS2, MRC1 and CD163 in the presence of IgG, BxPC-3 debris or both. Data are presented as the mean ± SD. **P < 0.01, ***P < 0.001, ns, no significance

Previously, we discovered IL-1β-producing M2 polarised TAMs in hepatocellular carcinoma; therefore, we investigated whether a similar phenomenon could be seen in PDAC. Consistent with previous studies,^13^ abundant M2 polarised TAMs were observed in human PDAC samples (Fig. 1a). To test the possible pro-inflammatory effects of M2 polarised macrophages induced by PDAC cell debris, which could be naturally formed or therapy-induced,^14^ we used an in vitro model of IL-4/IL-13-induced M2 polarised macrophages. As expected, IL-4 and IL-13 treatment of THP-1-derived macrophages showed typical characteristics of alternatively activated macrophages, with significantly reduced *NOS2* (nitric oxide synthase 2, iNOS) and increased *ARG1* (arginase 1) expression (Fig. 1b). Typical markers CD68, CD163, and CD206 were also confirmed using flow cytometry (Fig. 1c, superior panel). When these M2 polarised macrophages were stimulated with BxPC-3 cell-derived debris, a marked increase in IL-1β secretion was noted (Fig. 1d, left panel). A similar effect was observed when human monocyte-derived macrophages were tested (Fig. 1d, right panel), and the phenotype of alternatively activated macrophages was confirmed using flow cytometry (Fig. 1c, inferior panel). These results were further confirmed by immunoblotting analysis (Fig. 1d). To test whether the IL-1β production was caused by an M1 polarisation of macrophages upon cell debris stimulation, we detected the mRNA levels of phenotypic markers including *ARG1*, *NOS2*, *CD163* and *MRC1* (mannose receptor C-type 1). The expression levels of these markers were unchanged after cell debris treatment (Fig. 1f), suggesting no phenotypic alteration had occurred. Therefore, we inferred that PDAC cell debris was able to induce IL-1β secretion from M2 polarised macrophages, as observed in liver cancer.

### Cell debris induces IL-1β production in M2 polarised macrophages via TLR4-TRIF-NF-κB signalling

**Fig. 2:**
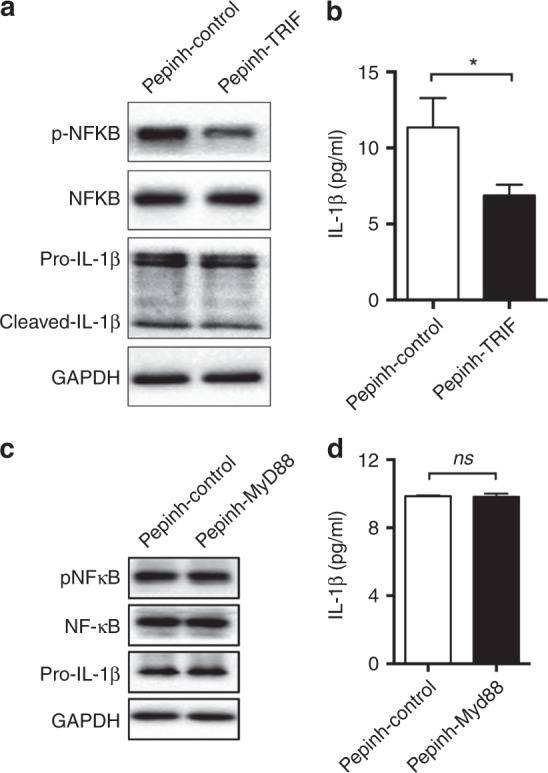
TRIF rather than MyD88 mediates cancer cell debris-induced IL-1β production. Following pre-treatment with (**a**, **b**) Pepinh-TRIF (5 µM, 6 h) or (**c**, **d**) Pepinh-MyD88 (5 µM, 6 h), THP-1-derived M2 polarised macrophages were activated by BxPC-3 debris for 24 h. (**a**, **c**) The levels of pNF-κB, NF-κB, and pro-IL-1β were determined using western blotting. (**b**, **d**) The release of IL-1β was determined using ELISA. Data are presented as the mean ± SD. *P < 0.05, ns, no significance

We then explored the mechanism by which cancer cell debris induces IL-1β production. In liver cancer we had shown that aberrant TLR-4-TRIF-NF-κB signalling mediated cancer cell debris-induced IL-1β production. However, whether this signalling pathway functions in other clinical scenarios was unknown. Intriguingly, a TRIF inhibitor was able to reduce pNF-κB levels and consequent IL-1β production (Fig. 2a, b). In contrast, inhibition of MYD-88, another common adaptor of TLR4, did not affect the levels of pNF-κB and IL-1β (Fig. 2c, d). Therefore, TLR-4-TRIF-NF-κB signalling might be a conserved mechanism mediating cancer cell debris-induced IL-1β production in M2 polarised macrophages.

### Expression of IgG in human PDAC and IgG-enhanced IL-1β production

**Fig. 3:**
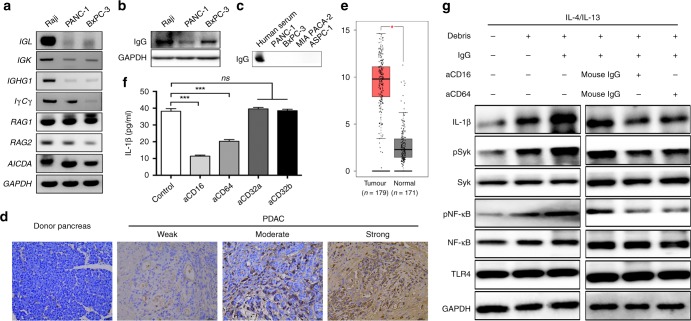
Expression of IgG in human PDAC and IgG-enhanced IL-1β production in M2 polarised macrophages. **a** The mRNA levels of *IGL*, *IGK*, *IGHG1*, *IγCγ*, *RAG1*, *RAG2*, and *AICDA* in PANC-1 and BxPC-3 cells were determined. Raji cells were used as a positive control. **b** The levels of IgG in PANC-1 and BxPC-3 cells were detected using western blotting. **c** The IgG level in the culture media of PDAC cell lines was analysed using western blotting. Human serum was used as a positive control. **d** Immunohistochemical staining showing IgG expression in PDAC tissue and donor-derived pancreases. Scale bar represents 25 µm. **e** The *IGHG1* mRNA level in PDAC tumour and paratumoural tissue from the TCGA database. **f** THP-1-derived M2 polarised macrophages were incubated with anti-CD16, anti-CD32a, anti-CD32b, or anti-CD64 antibodies, followed by stimulation with BxPC-3 debris and IgG. The IL-1β level was determined using ELISA. **g** THP-1-derived M2 polarised macrophages were pre-treated with anti-CD16 or anti-CD64 antibodies and stimulated with BxPC-3 debris in the presence or absence of IgG for 24 h. The levels of pSYK, SYK, pNF-κB, NF-κB,IL-1β, and TLR4 were determined using western blotting. Data are presented as the mean ± SD. *P < 0.05, ***P < 0.001, ns, no significance

Intriguingly, we observed enhanced IL-1β production in the presence of IgG when THP-1-derived macrophages or monocyte-derived macrophages were exposed to BxPC-3 cell debris (Fig. 1c, d), indicative of a special role of IgG in pro-inflammatory M2 polarised macrophages. To evaluate IgG expression in human PDAC, we first checked its expression in frequently used PDAC cell lines, including PANC-1 and BxPC-3. The mRNA encoding the IgG heavy chain constant region (*IGHG1*) and immunoglobulin light chains (IgK (*IGK*) and Igλ *(IGL*)) were detected (Fig. 3a). Moreover, amplification of *RAG1* (encoding recombination activating 1), *RAG2* (encoding recombination activating 2), AICDA (encoding activation induced cytidine deaminase), and *IγCγ*, which are critical for V(D)J recombination and somatic hypermutation of immunoglobulin, was also observed in both cell lines (Fig. 3a). Consistently, production of the whole molecule IgG (~70 kDa) was also detected (Fig. 3b). However, no positive bands were observed in the supernatants of PDAC cell lines (Fig. 3c), suggesting minimal secretion of the IgG. For further validation, we investigated the IgG expression in the human pancreas and in 48 PDAC tissues. While tumours displayed different levels of IgG (21/48 weak, 9/48 moderate and 18/48 strong), lower levels were observed in the healthy pancreas (Fig. 3d). In addition, increased expression of *IGHG1* was found in a large panel of human PDAC tissues (Fig. 3e). These findings indicated the wide expression of IgG in human PDAC.

To investigate the mechanism by which IgG amplifies IL-1β production induced by cancer cell debris in M2 polarised macrophages, we blocked the Fc gamma receptors (FcγR) using anti-CD16, anti-CD32a, anti-CD32b, and anti-CD64 antibodies. Only anti-CD16 and anti-CD64 antibodies were able to partially reduce IL-1β production (Fig. 3f), suggesting that FcγRI/III plays a critical role in IgG-enhanced IL-1β production. To further confirm this result, we observed significantly reduced levels of phosphorylation of SYK, an essential protein tyrosine kinase for FcγR signalling in macrophages,^15^ in the presence of anti-CD16 or anti-CD64 antibodies (Fig. 3g). Notably, p-NF-κB and IL-1β levels were also downregulated in case of CD16 or CD64 blockade. Thus, IgG-induced activation of FcγRI/III-SYK signalling appeared to enhance NF-κB signalling and promote IL-1β production in the presence of cancer cell debris.

### IL-1β induces EMT and metastasis in PDAC

**Fig. 4:**
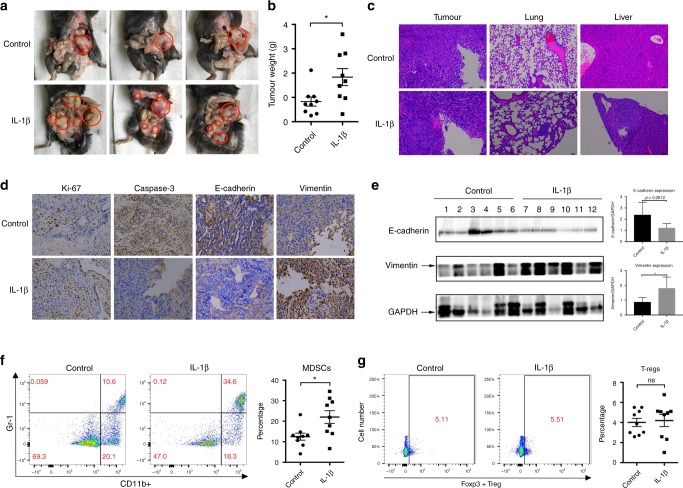
IL-1β promotes PDAC metastasis in vivo. Mice with orthotopic pancreatic cancer were treated intra-peritoneally with IL-1β (500 ng per week, 5 weeks) or vehicle. Gross peritoneal metastasis was examined. Red circles indicate the primary or metastatic tumours. **a** Three representative mice in each group are shown. **b** Tumour weights were analysed (*n* = 9). **c** Lung and liver metastases were analysed using H&E staining. Scale bar represents 25 µm. **d** Immunohistochemical staining of the indicated markers was performed. Scale bar represents 25 µm. **e** Immunoblotting of the indicated markers was performed in primary tumours from both the IL-1β or control groups. The relative levels of vimentin and E-cadherin are presented as the mean ± SD. **f** MDSCs and CD4^+^ T regulatory cells (**g**) in the tumours were analysed using flow cytometry. Data are presented as the mean ± SD. *P < 0.05, ns, no significance

To understand the biological effects of increased IL-1β release in PDAC, we injected murine IL-1β intraperitoneally into an orthotropic PDAC mouse model. Compared with that in the PBS control, IL-1β caused increased peritoneal metastases and a higher tumour burden (Fig. 4a, b). Distant metastasis to the lung and liver was also observed in the IL-1β-treated mice but not in the PBS-treated mice (Fig. 4c and Table 1). When checking the primary tumour, we found increase levels of Ki-67 and vimentin, as well as decreased levels of caspase-3 and E-cadherin (Fig. 4d). Increased vimentin and decreased E-cadherin were confirmed by further immunoblotting (Fig. 4e). These results suggested inhibition of apoptosis and enhanced proliferation and epithelial-mesenchyme transition (EMT) of the cancer cells. We further identified an immunosuppressive microenvironment within the tumour, showing increased recruitment of myeloid-derived suppressor cells (MDSCs) rather than regulatory CD4^+^ T cells (Tregs) (Fig. 4f, g). These results indicated that IL-1β could promote tumour progression in multiple ways.

**Table 1:** Metastatic status of the orthotropic models

	Control (*n* = 9)	IL-1β (*n* = 9)	*P* value
Lung metastasis	0	5	0.029
Liver metastasis	0	1	0.471

**Fig. 5:**
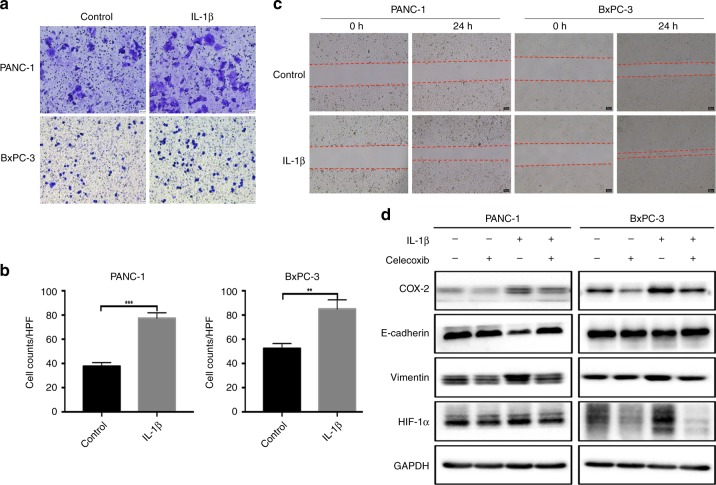
IL-1β induces EMT in PDAC cells in vitro. **a**–**c** PANC-1 and BxPC-3 cells were treated with IL-1β (1 ng/ml). The migratory capacity was analysed by Transwell assays (**a**, **b**) and wound healing assays (**c**). Scale bar represents 25 µm. **d** PANC-1 and BxPC-3 cells were treated with IL-1β (1 ng/ml, 24 h) with or without celecoxib pre-treatment (5 µM, 12 h). The levels of COX-2, E-cadherin, vimentin, and HIF-1α were measured using western blotting. Data are presented as the mean ± SD. **P < 0.01

We further tested the role of IL-1β in human PDAC cell lines. IL-1β significantly enhanced the migration capacity of both PANC-1 and BxPC-3 cells (Fig. 5a–c). Similar to liver cancer, IL-1β induced EMT in PDAC cell lines accompanied by COX-2 and HIF-1α overexpression (Fig. 5d). According to the literature and our previous study,^16^ we hypothesised that COX-2-HIF-1α signalling mediated IL-1β-induced EMT through the IL-1 receptor. Indeed, a COX-2 selective inhibitor, celecoxib, reversed IL-1β-induced EMT in these cells (Fig. 5d), suggesting a potential role of celecoxib in PDAC treatment.

### Celecoxib synergises gemcitabine to treat PDAC

**Fig. 6:**
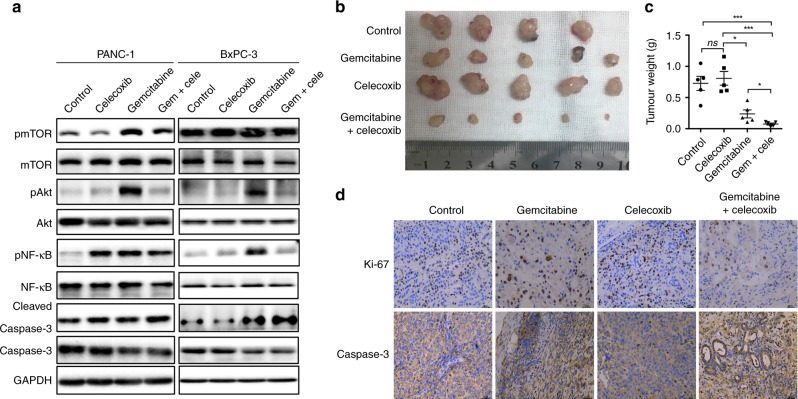
Celecoxib synergises gemcitabine during PDAC treatment. **a** PANC-1 and BxPC-3 cells were treated with vehicle, celecoxib, gemcitabine, or gemcitabine plus celecoxib. The levels of pmTOR, mTOR, pAKT, AKT, pNF-κB, NF-κB, caspase-3 and cleaved caspase-3 were determined using western blotting. **b**–**d** Orthotopic pancreatic cancer mice were treated as indicated for two weeks. **b** Tumours are shown. **c** Tumour weights were analysed (*n* = 5). **d** The expression of the indicated proteins was analysed using immunohistochemical staining. Scale bar represents 25 µm. Data are presented as the mean ± SD. *P < 0.05, ***P < 0.001, ns, no significance

Gemcitabine is a widely used chemotherapeutic drug for human PDAC.^17^ PDAC cells exposed to gemcitabine displayed increased levels of p-mTOR, p-AKT, and p-NF-κB (Fig. 6a), which are important for drug resistance in PDAC.^18,19^ However, treatment with celecoxib downregulated the activation of mTOR, AKT, and NF-κB, suggesting a sensitisation role of celecoxib in PDAC chemotherapy. To verify the therapeutic role of this combination, we performed three independent experiments using an allograft mouse model. The combination of gemcitabine and celecoxib demonstrated better tumour control compared with that of gemcitabine or celecoxib alone (Fig. 6b, c). Immunohistochemical staining revealed fewer Ki-67 positive cells, although caspase-3 levels looked similar in all tumour cells (Fig. 6d). These results showed that gemcitabine plus celecoxib is a promising combination strategy to treat PDAC.

## Discussion

Although the relationship between inflammation and tumours is well documented, few achievements have been successfully translated into the clinic to date. In the microenvironment of solid tumours, TAMs are key players that regulate inflammation. However, despite decades of research, the roles of these versatile cells are incompletely understood. The phenotype and function of TAMs can change rapidly between two extremes (i.e., pro-inflammation and anti-inflammation); therefore, it is challenging to capture an accurate picture of TAMs functions. Currently, the dissociation of phenotype and function in macrophages has been revealed in a few diseases, such as rheumatic arthritis and tumours. In the present study, we reported the detection of IL-1β-producing M2 polarised macrophages in pancreatic cancer, and revealed that their pro-inflammatory function could be further enhanced by IgG via crosstalk between the TLR4-TRIF and FcγRI/III-SYK signalling pathways. The demonstration of a potent pro-tumour effect of IL-1β in PDAC allowed us to hypothesise that interference with IL-1 receptor signalling in cancer cells by suppressing COX-2 could be a promising method to sensitise PDAC cells to gemcitabine. Thus, an anti-inflammatory strategy in the chemotherapy of PDAC could be valuable, although further studies are needed to confirm this hypothesis.

Unlike other solid tumours, PDAC is characterised by poor vascularization and hypoxia. Micronecrotic regions are found frequently in PDAC. Cancer cell debris and cancer cell-derived IgG can exist surrounding these regions. Noticeably, IgG is not likely to be secreted by B-lymphocytes or PDAC cells because we did not detect significant IgG in the supernatant of PDAC cell lines. Thus, in case of PDAC at least, IgG is believed to be released extensively when cancer cells die from necrosis. The simultaneous access to both necrotic cell debris and IgG seems particular to PDAC, because IgG originating from blood is largely limited by the tumour’s poor vascularisation.

IL-1β is a very potent cytokine that can induce EMT in various types of cancer cells.^20^ In particular, IL-1β was reported to promote the invasiveness of pancreatic cancer.^21^ We used in vitro and in vivo models to show that IL-1β also induced EMT in PDAC. In addition, IL-1β could lead to an immunosuppressive microenvironment in both primary tumours and metastatic distant organs (unpublished data). Although M2 polarised macrophages were proven to induce EMT through TLR4-IL-10 signalling in PDAC,^22^ the results of the present study provided another mechanism by which M2 polarised macrophages could induce EMT in PDAC, i.e., by secreting IL-1β, suggesting a complicated role of alternatively activated TAMs in PDAC.

Given the pro-tumoural effect of IL-1β, it would be reasonable to test blockade of IL-1 receptor signalling in cancer cells as a possible treatment for PDAC. Several targets in the IL-1 receptor signalling pathway could be chosen. For instance, canakinumab, a human anti-IL-1β monoclonal antibody that was shown recently to be associated with a reduced incidence of lung cancer,^23^ could be used to neutralise IL-1β in the tumour microenvironment. Anakinra (a receptor antagonist for IL-1R1), rilonacept (soluble IL-1 receptor inhibitor), or other IL-1β blocking methods could also be considered.^24^ Targeting IL-1β is relatively safe because the human body does not need it frequently unless infected with pathogens. In the present study, we used celecoxib, a widely used non-steroidal anti-inflammatory drug, to inhibit HIF-1α synthesis and consequent EMT induction. While celecoxib itself showed no treatment efficacy in vivo, it enhanced the anti-tumoural effects of gemcitabine, probably by reversing acquired drug resistance mediated by mTOR, AKT, and NF-κB.

Several groups have used celecoxib to treat solid cancers in clinical trials, showing contradictory results. Celecoxib failed to improve survival in patients with advanced non-small-cell lung cancer, metastatic prostate cancer, and HER2-negative breast cancer.^25–27^ However, celecoxib reduced tumour recurrence in colorectal adenomas.^28^ A meta-analysis showed that celecoxib was associated with better treatment responses in several types of cancer.^29^ However, most of these trials did not select patients using COX-2 or IL-1β levels. Inflammation is also important for immune attack by T lymphocytes; therefore, anti-inflammation treatment is not reasonable for cancers with good responses to immunotherapy. In PDAC, however, immune cells may be less important compared with other tumours with an ample blood supply, and anti-inflammation treatment might be more valuable in PDAC chemotherapy, especially for tumours with necrotic regions. Although a phase II trial testing this strategy failed in PDAC,^30^ the failure might have been related to the small number and late stage of the patients. Other similar studies showed promising results.^31,32^ Further trials are currently ongoing, and we believe that selection of patients with indications of a significant inflammatory tumour microenvironment is crucial for the success of the trials.

In conclusion, we demonstrated M2 polarised macrophage-mediated IL-1β production in PDAC and demonstrated that a crosstalk between the TLR4-TRIF and FcγRI/III-SYK signalling pathways were critical for IL-1β production. In addition, we showed that IL-1β promoted EMT and metastasis of PDAC cells, and that the use of celecoxib could sensitise tumour cells to gemcitabine. However, further studies are needed to identify suitable patients.

## Supplementary information

Supplementary Tables

## Data Availability

All data and materials were available on line. https://pan.baidu.com/s/12xE-MT6oF1ZdS4rMGfaNzA.
