# Supplementary material for: Tumour cell-derived debris and IgG synergistically promote metastasis of pancreatic cancer by inducing inflammation via tumour-associated macrophages
Source: Br J Cancer. 2019 Oct 7;121(9):786–95. doi: 10.1038/s41416-019-0595-2 (PMC6889176; doi:10.1038/s41416-019-0595-2)
Supplement: Supplementary file 1 — Supplementary Tables [file 41416_2019_595_MOESM1_ESM.docx]

**Supplementary Tables**

Table S1 Primers for reversed transcription PCR

| Gene name | Primer sequence 5’-3’ | Annealing temperature |
| --- | --- | --- |
| IGHG1 | GACTGGCTGAATGGCAAGGAG (sense)  GGCGATGTCGCTGGGATAGAA (antisense) | 56℃ |
| Igκ | TGAGCAAAGCAGACTACGAGA (sense)  GGGGTGAGGTGAAAGATGAG (antisense) | 54℃ |
| AID | External GAAGAGGCGTGACAGTGCT (sense)  CGAAATGCGTCTCGTAAGT (antisense)  Internal CCTTTTCACTGGACTTTGG (sense)  TGATGGCTATTTGCACCCC (antisense) | 54℃  52℃ |
| RAG1 | External TGGATCTTTACCTGAAGATG (sense)  CTTGGCTTTCCAGAGAGTCC (antisense)  Internal CACAGCGTTTTGCTGAGCTC (sense)  AGCTTGCCTGAGGGTTCATG (antisense) | 52℃  54℃ |
| RAG2 | External TGGAAGCAACATGGGAAATG (sense)  CATCATCTTCATTATAGGTGTC (antisense)  Internal TTCTTGGCATACCAGGAGAC (sense)  CTATTTGCTTCTGCACTG (antisense) | 52℃  52℃ |
| Iγ-Cγ | External GGGCTTCCAAGCCAACAGGGCAGGACA (sense)  CAAGCTGCTGGAGGGCACGGT (antisense)  Internal GGTGAACCGAGGGGCTTGT (sense)  CGCTGCTGAGGGAGTAGAGT (antisense) | 54℃  52℃ |
| Igλ | GAGCCTGACGCCTGAG (sense)  ATTGAGGGTTTATTGAGTGCAG (antisense) | 54℃ |
| GAPDH | GGGAGCCAAAAGGGTCATCATCTC(sense)  CCATGCCAGTGAGCTTCCCGTTC (antisense) | 57℃ |

Table S2 Primers for real time PCR

| Gene name | Forward (5’ to 3’) | Reverse (5’ to 3’) |
| --- | --- | --- |
| IL-1β | AAAGCTTGGT GATGTCTGGTC | GGACATGGAGAACACCACTTG |
| NOS2 | TCATCCGCTATGCTGGCTAC | CCCGAAACCACTCGTATTTGG |
| Arg-1 | GGGTTGACTGACTGGAGAGC | CACATCACACTCTTGTTCTTTAAGT |
| ß-actin | TGGCACCCAGCACAATGAA | CTAAGTCATAGTCCGCCTAGAAGCA |
| CD163 | TTTGTCAACTTGAGTCCCTTCAG | TCCCGCTACACTTGTTTTCAG |
| MRC1 | GGGTTGCTATCACTCTCTATGC | TTTCTTGTCTGTTGCCGTAGTT |
